# Exploring What Factors Mediate Treatment Effect: Example of the STarT Back Study High-Risk Intervention

**DOI:** 10.1016/j.jpain.2016.08.005

**Published:** 2016-11

**Authors:** Gemma Mansell, Jonathan C. Hill, Chris Main, Kevin E. Vowles, Daniëlle van der Windt

**Affiliations:** ∗ Research Institute for Primary Care and Health Sciences, Keele University, Keele, Staffordshire, United Kingdom; † Department of Psychology, University of New Mexico, Albuquerque, New Mexico

**Keywords:** Mediation analysis, low back pain, psychological intervention

## Abstract

Interventions developed to improve disability outcomes for low back pain (LBP) often show only small effects. Mediation analysis was used to investigate what led to the effectiveness of the Stratified Targeted Treatment (STarT) Back trial, a large primary care-based trial that treated patients consulting with LBP according to their risk of a poor outcome. The high-risk subgroup, randomized to receive either psychologically-informed physiotherapy (n = 93) or current best care (n = 45), was investigated to explore pain-related distress and pain intensity as potential mediators of the relationship between treatment allocation and change in disability. Structural equation modeling was used to generate latent variables of pain-related distress and pain intensity from measures used to identify patients at high risk (fear-avoidance beliefs, depression, anxiety, and catastrophizing thoughts). Outcome was measured using the Roland–Morris Disability Questionnaire. Change in pain-related distress and pain intensity were found to have a significant mediating effect of .25 (standardized estimate, bootstrapped 95% confidence interval, .09–.39) on the relationship between treatment group allocation and change in disability outcome. This study adds to the evidence base of treatment mediation studies in pain research and the role of distress in influencing disability outcome in those with complex LBP.

**Perspective:**

Mediation analysis using structural equation modeling found that change in pain-related distress and pain intensity mediated treatment effect in the STarT Back trial. This type of analysis can be used to gain further insight into how interventions work, and lead to the design of more effective interventions in future.

## Highlights

• Identifying how treatments work is important in making them more effective in the future.
• Changes in psychological distress and pain intensity are mediators of treatment effect.
• This study adds to the evidence base for the role of mediators in pain research.

Low back pain (LBP) has a global point prevalence of 9.4% and prevalence of 15% in Western European countries.^23^ In the United Kingdom, LBP has been reported to be the most common reason for patients consulting their general practitioner.^24^ LBP has a wide impact, not only on the sufferer but also on health care costs.^22^, ^32^, ^34^ workplace absence,^32^, ^55^ and social support.^34^ Although the prognosis of many patients with LBP who consult is good,^35^, ^38^ there is variation among individuals and longer-term problems with pain and disability are often reported up to a year after consultation.^17^, ^35^ Identifying factors that are associated with long-term disability has been the focus of recent research, and evidence has been found for a number of psychological factors being predictive of outcome (eg, fear-avoidance beliefs,^28^, ^39^ catastrophizing thoughts,^14^ and depression^14^, ^28^).

Treatments to improve disability by targeting these factors, such as cognitive-behavioral therapy (CBT), have been shown to be successful in secondary care populations^10^, ^21^ but mixed results have been reported in primary care populations, possibly because of the more heterogeneous population and the less intensive psychological interventions provided.^53^ One potential solution that has been recently explored is the idea of providing stratified care on the basis of a person's risk of a poor outcome. A key example of this, the Stratified Targeted Treatment (STarT) Back approach,^20^ was reported to be effective at reducing disability when patients consulting primary care for LBP were assessed for their risk of a poor outcome and matched to an intervention on the basis of their risk profile. Patients with the most complex problems (a score of ≥4 on the tool, with this score incorporating ≥4 items on a psychological subscale) received specialized, psychologically informed physiotherapy treatment to address these factors. However, although the factors chosen to help stratify patients were known prognostic factors, this does not necessarily mean that these same factors were also strong targets for treatment in this group (ie, were mediating factors).

The purpose of this study was to investigate if the psychological factors used to stratify patients into the high-risk group mediated treatment outcomes in patients who were then given treatment designed to address those factors. Because mediators help explain how treatment achieves its effect and identify factors that can be modified by treatment,^30^ such an analysis of the STarT Back high-risk group would allow us to test the hypothesis that these psychological factors were indeed associated with the effectiveness of the treatment at 4-month follow-up.

## Methods

### STarT Back Trial

The STarT Back trial (ISRCTN37113406) was a randomized controlled trial that compared stratified care with current best care in primary care patients with LBP.^20^ At baseline, 851 patients were randomized (568 to intervention and 283 to control), with a mean age of 50 (SD = 14.8) years. Of this population 58.8% were female and 41.2% were male. In the high-risk group specifically, the mean age was slightly higher (54, SD = 12.88 years) and a slightly lower percentage were female (56.5%). Pain duration in this group was reported as <1 month (15.9%); 1 to 3 months (2.3%); 4 to 6 months (16.7%); 7 months to 3 years (21.7%); and >3 years (25.4%). Patients were included in the trial if they were aged 18 years or older, could speak English, and had LBP of any duration, with or without associated radiculopathy. Patients were excluded if their pain was potentially indicative of a serious disorder (eg, cancer), if they had serious comorbidities that would negatively affect treatment (eg, schizophrenia), were pregnant, or were undergoing other forms of treatment.^20^ If patients were assigned to stratified care, treatment was matched to the patient's prognostic risk using a brief prognostic index, the Keele STarT Back tool,^19^ which consisted of 9 questions relating to 8 physical and psychological factors known to be predictive of persistent LBP-related disability. Scores on the overall tool score and a 5-item psychological subscale score allocated patients to low-, medium-, or high-risk targeted treatment groups. A score of ≥4 on the psychological subscale specifically indicated the presence of symptoms of pain-related distress, meaning the patient was typically more complex to treat and therefore at higher risk of a poor treatment outcome. These high-risk patients went on to receive psychologically informed physiotherapy^31^ delivered by physiotherapists who had undergone 6 days of training sessions focused on skills to help them address psychosocial barriers to recovery. The psychological factors discussed as part of this training included those measured in the STarT Back tool such as fear-avoidance beliefs, catastrophizing thoughts, anxiety, and depression. However, although the high-risk training was on the basis of a cognitive-behavioral framework, the training did not constitute full-blown cognitive-behavior therapy (which would have required much more intensive training), but aimed to establish psychologically informed practice, in which physiotherapists were confident to address patients' unhelpful beliefs, emotions, and behavioral responses to pain.^31^ The individual psychological constructs identified by the STarT Back tool were therefore not systematically addressed, but instead addressed as and when they presented in a patient. Therapists were given skills to reduce pain-related distress through clear communication, reassurance, and activity promotion to improve physical function. It therefore seemed appropriate to test whether the treatment effect on physical function was mediated by changes in overall pain-related distress rather than through individual psychological factors. Ethical approval and informed consent from study participants was gained in the original study and additional consent was therefore not required for the present study.

### Measures

#### LBP-Specific Disability

The primary outcome in the STarT Back trial was back pain disability at 12 months measured using the Roland-Morris Disability Questionnaire (RMDQ).^41^ In the present study, RMDQ score at baseline and 4-month follow-up was included. The RMDQ is a LBP-specific measure comprised of a list of 24 statements related to the ability to carry out movements or everyday activities. Higher scores indicate greater back pain-related disability. The RMDQ has been reported to have good psychometric properties overall in LBP populations.^16^, ^37^, ^40^, ^41^, ^44^, ^49^

#### Pain-Related Distress

The psychological variables included in the screening tool for which full measures were available were included as potential mediators: catastrophizing (Pain Catastrophizing Scale; PCS^50^), fear-avoidance beliefs (Tampa Scale for Kinesiophobia; TSK^26^), and anxiety and depression (Hospital Anxiety and Depression Scale, HADS^56^). Each of these measures captured an aspect of pain-related distress, which was tested via factor analysis (see Supplementary Section 1). The PCS is made up of 13 items each scored on a 5-point Likert scale, with a higher total score signifying greater pain catastrophizing. The 17-item, unidimensional version of the TSK was used in the STarT Back study. A higher score on this measure indicates more severe fear-avoidance beliefs. The HADS contains 14 items, with 7 items each for anxiety and depression. These are scored on a 4-point Likert scale, with a higher score indicating greater anxiety and/or depression. Each of the measures are used frequently in primary care musculoskeletal research, and have been reported to have good measurement properties in this population.^4^, ^9^, ^54^ There is some debate about the psychometric properties of the TSK, including its factor structure (see French et al^12^ and Lundberg et al^29^ for reviews), but use of the TSK as a unidimensional tool is common. Within the present population, all of the measures were reported to have good internal consistency with baseline Cronbach α values of >.70 (.94 for the PCS; .73 for the TSK; .82 and .85 for the HADS subscales of anxiety and depression, respectively).

#### Pain Intensity

Pain intensity is often used as an outcome measure but is also known to have an important role in other patient outcomes^36^ and is also strongly related to psychological factors.^52^ In the original STarT Back trial,^20^ pain intensity was not specifically the focus of the high-risk intervention, but in primary care settings in which many patients consult for musculoskeletal pain, pain is often the focus of treatment. This variable was therefore examined as an additional potential mediating factor alongside the psychological factors. Three measures of pain intensity were available in the STarT Back data set; least pain over the past 2 weeks, average pain over the past 2 weeks, and pain intensity on the day the questionnaire was completed. Each of the variables were measured on an 11-point Likert scale, with a higher score indicating higher pain intensity.

### Mediation Analysis Using Structural Equation Modeling

Mediation analysis was carried out using structural equation modeling (SEM), which combines linear regression and factor analysis^51^ and maps out the paths between observed and unobserved variables and the error associated with each variable.^5^ SEM is a useful technique for performing mediation analysis because it accounts for error in the observed variables and can test more complex models than traditional regression techniques. This type of analysis requires multiple variables or items per factor (latent variable), which allows the factor to be measured with greater reliability.^1^, ^48^

### Statistical Analysis

The analysis of mediating factors is complex and a number of steps were required to conduct this analysis. All analyses were conducted using SPSS PASW statistics package version 18 (SPSS Inc, Chicago, IL) and AMOS (add-on statistics package to SPSS) version 19.

Descriptive statistics (means and SDs) were calculated for baseline and 4-month follow-up for the outcome and potential mediator variables for each of the 3 prognostic subgroups, and distributions checked for normality because this is an assumption underlying all of the analyses. Descriptive statistics were also used to examine baseline characteristics of participants responding versus not responding at 4-month follow-up.

#### Creation of Residualized Change Scores

Because change was of interest in the present analysis, residualized change scores were calculated for each of the potential mediator and outcome variables. Residualized change scores represent the difference between the score at follow-up compared with what was predicted at baseline,^49^ thereby controlling for baseline score. Residualized change scores are frequently used in studies of mediation^15^, ^43^ and are calculated by running a linear regression with the follow-up score as the outcome and the baseline score as the predictor, and saving the residual values (difference between the observed value at follow-up and the value predicted at baseline), which were then used in all subsequent analyses.

#### Testing Criteria for Potential Mediation

**Figure 1:**
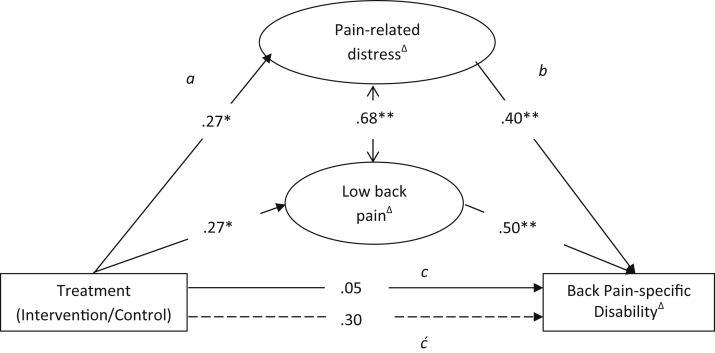
Full SEM model for mediating effect of changes in pain-related distress and pain intensity on change in disability: high-risk group (full information maximum likelihood; n = 236). ^Δ^Residualized change. *P < .05. **P < .01. All values are standardized.

For a variable to be a potential mediator of outcome, it must be potentially modifiable (ie, change over time) and it must be associated with treatment and outcome (the *a* and *b* paths in Fig 1). To examine modifiability, the absolute change that occurred between baseline and 4-month follow-up was calculated and examined. To examine the associations on the *a* and *b* paths, linear regression analyses were performed to investigate the relationships between treatment group allocation (intervention or control) and residualized change in each of the potential mediators, and between residualized change in each of the potential mediators and residualized change in outcome.

#### Creation of Latent Variables

To create latent variables to be used in the SEM, exploratory factor analysis (EFA) and subsequently confirmatory factor analysis (CFA) were conducted with the 4 psychological mediators to ascertain whether the different measures represented a single ‘pain-related distress’ factor, and an EFA was also conducted on the pain intensity measures to ascertain whether they represented a single factor of ‘LBP.’ In SEM, relationships between variables of interest must be tested to ensure they are correctly specified to have (or not have) a relationship in the model.^42^ CFA was therefore also used to confirm whether the 2 latent variables of pain-related distress and LBP were representative of distinct latent variables. A strong correlation (ie, ≥.60) would need to be acknowledged in the model by means of a double-headed arrow to show covariance.

The factor analyses were performed on the entire STarT Back sample rather than only the high-risk group reported in this article, and are not reported fully in this article (see Supplementary Section 1 for a summary of the results). This was to ensure that the number of cases was adequate for factor analysis to be performed. The mediation analysis was only performed on the high-risk group.

#### Mediation Analysis

The statistical interpretation of mediation analysis can be broken down into separate effects (Fig 1). The *c* path is the direct effect of treatment on outcome, before taking into account the effects of specific mediating variables. Paths *a* and *b* make up the mediating pathway, with the mediating effect usually being described in the literature as the product of coefficients (*ab*).^30^ The *ć* path denotes the total effect of the whole model (*ab* + *c*). SEM provides the product of coefficients of the mediating effect with bias-corrected bootstrapped confidence intervals (CIs), which is currently seen as an optimal way of performing mediation analysis.^13^, ^18^ One thousand bias-corrected bootstrapped samples and 95% CIs were used in the present analysis. Complete case data were used for this analysis because the bias-corrected CIs can only be generated with complete data, but a sensitivity analysis using all available data (full information maximum likelihood; n = 236) was also conducted as a comparison (Appendix 1).

The following goodness of fit indices were used to assess how well the proposed model fitted the available data: χ^2^ statistic, χ^2^/df, comparative fit index (CFI), root mean square error of approximation (RMSEA), and standardized root mean square residual (SRMR).^3^, ^5^ Good or adequate model fit is indicated by a nonsignificant χ^2^ value, a χ^2^/df of between 2 and 5, a CFI of ≥.95 and RMSEA and SRMR values of ≥.08. No single fit index is seen as superior, and it is therefore recommended that judgement of fit is on the basis of the overall assessment of several indices.^6^

## Results

### Testing Criteria for Potential Mediation

Table 1 contains mean scores at baseline and mean change at 4-month follow-up for all of the potential mediator variables. There was a difference between the amounts of change in potential mediators observed in the treatment groups at 4-month follow-up compared with the control group, including a 4.4-point difference in change for catastrophizing thoughts and a 5.8-point difference in change for fear-avoidance beliefs, representing the largest changes. The SDs for all mediators were quite large, suggesting large variability in change within the high-risk group. Tests for normality (skewness and kurtosis values, histograms and p-plots) indicated some departure from normality with values >1.0 for several variables.

After calculating residualized change scores, linear regression analyses for each of the potential mediator variables with disability outcome were performed (Table 2). The results indicated that in the treatment group, residualized change in the psychological variables strongly predicted residualized change in RMDQ, accounting for 25–39% of the variance in this outcome. Residualized change in the pain variables were shown to be stronger predictors; they accounted for between 51% and 63% of the variance of residualized change in RMDQ. In the control group, residualized change in all potential mediators also accounted for a large amount of variance of residualized change in RMDQ. This showed support for the psychological factors as well as the pain variables to potentially mediate treatment outcome, because these variables do show change over time and are associated with outcome (*b* path).

Finally, it was important to also test the *a* path, or the relationship between treatment allocation and residualized change in each of the potential mediators. If the treatment had little effect on the potential mediating variables then the variables are unlikely to be the mechanism through which the treatment was successful. The results in Table 3 show that a small proportion of variance (between 2% and 12%) of residualized change was explained by treatment allocation (stratified care vs control) for each of the potential mediators. For residualized change in anxiety in particular, the association was very weak and not statistically significant, as indicated by the 95% CI. The results indicated that treatment allocation had the strongest association with residualized change in fear-avoidance beliefs.

In summary, these preliminary analyses show support for the psychological and pain variables to be potential mediators of the effects of the high-risk treatment. The variables changed significantly between baseline and follow-up, and were associated with residualized change in outcome (disability). Allocation to the high-risk treatment arm was found to be predictive of residualized change in all of the potential mediator variables, with the exception of anxiety. The EFA and CFA (Supplementary Section 1) confirmed that the 4 psychological factors were representative of a pain-related distress latent variable, which was distinct from a pain intensity latent variable represented by 3 pain measures. However the 2 latent variables were found to be strongly correlated. These variables were therefore taken forward as planned as mediating pathways of the relationship between treatment allocation and residualized change in functional outcome.

#### Mediation Analysis

The mediation model for residualized change in pain-related distress and pain intensity as mediators of the relationship between allocation to the high-risk STarT Back treatment and residualized change in disability is shown in Fig 1. The strong correlation found between the latent variables of pain-related distress and pain intensity is represented by an arrow indicating covariance. Considering all model fit statistics the model was judged to provide adequate fit to the data (χ^2^ = 54.36_23_, *P* < .05, χ^2^/df = 2.36, CFI = .96, RMSEA = .10 (95% CI, .07–.13), SRMR = .05).

The *a* path in this model is interpreted as an average treatment effect, because of the treatment allocation variable being binary (control = 0).^11^ The value of .27 for the *a* path between treatment allocation and pain-related distress therefore can be interpreted as the change in pain-related distress between baseline and 4-month follow-up being .27 units higher in the treatment group than in the control group (a larger change). Similarly, the *b* path of .40 can be interpreted as that a 1-unit change in pain-related distress leads to a .40 change in disability. The effects of the model (Table 4) show that the total effect (*ć*) of the model is .30. When the mediator variables (combined for pain-related distress and LBP) were added to the model the *ab* pathway explained a considerable proportion of the treatment effect; a statistically significant mediating effect of change in the latent variables was found (standardized indirect effect = .25, bootstrapped 95% CI, .09–.39).

The sensitivity analysis using all available data resulted in weaker coefficients for the *a* and *c* paths (.04 and .20, respectively) compared with the complete case analysis (.27 and .27, respectively), and a very small total effect (−.02) was found compared with the total effect of .30 in the complete case analysis. These results suggest that those who did not respond at follow-up were different to those who responded, in that they experienced a smaller change in the measures of pain-related distress and a slightly smaller change in physical function. The change in direction of the coefficients (negative direct and total effects for all sensitivity analysis) could also be indicative of a larger change in the control group when all available data were used. Nonresponse analysis indicated there were baseline differences between responders and nonresponders at 4-month follow-up, with responders being older, less disabled, and having lower scores for fear-avoidance beliefs, catastrophizing, and depression.

## Discussion

The STarT Back trial was originally designed to test whether a model of stratified care consisting of targeted treatment matched to prognostic subgroups would lead to improved patient outcomes compared with best current primary care for back pain. The aim of the present study was to test the hypothesis that the observed favorable outcomes of the stratified intervention in the STarT Back high-risk group were mediated by changes in pain-related distress and pain intensity.

The preliminary analyses showed that residualized change in 4 of the psychological variables used in the STarT Back trial and pain intensity met the criteria for potential mediation of treatment outcome. The mediation model confirmed the study hypothesis, with residualized change in pain-related distress and pain intensity being found to be mediators of the relationship between treatment group allocation and residualized change in disability.

### Comparison With Conceptual Model

When evaluating mediation models, it has been proposed that the paths can be split into action theory (*a* path) and conceptual theory (*b* path).^7^, ^8^ Action theory is described as the intervention's power to detect the potential mediator whereas conceptual theory is described as the potential mediator's power to detect the outcome.^7^ It has been suggested that these 2 elements form the theoretical basis between the 2 paths.^47^ If the association between the intervention and the potential mediator is weak, this suggests that the action theory has failed; the intervention is not doing enough to affect the mediator.^7^ If the association between the potential mediator and the outcome is weak, then the conceptual theory has failed; the intervention is targeting the wrong factors for change,^7^ meaning that the underlying theory is wrong. In the present analysis, this interpretation would suggest that the stronger associations between change in the mediators and change in disability (conceptual theory) show that targeting pain-related distress and pain intensity was important, but the weaker associations between treatment allocation and change in the mediators (action theory) suggest that the psychologically informed physiotherapy did not greatly influence these factors. This possibly reflects the fact that this treatment, although targeting psychological factors, did not target them as specifically or effectively as a more intensive treatment delivered by psychologists, such as CBT. This could also be because change occurred in the treatment and control groups, with the magnitude of this change sometimes being larger in the control group. Pain-related distress is only one of many potential mediators that could be explaining the treatment effect seen in the high-risk patients in the trial, and it is likely that other, unmeasured variables could have a stronger association with outcome, especially in the treatment group.

The alternative pathway to treatment outcome, through a change in LBP intensity, was found to be a stronger mediator of the relationship between the high-risk treatment and disability than change in pain-related distress. The 2 latent variables of pain-related distress and LBP were found to have a strong relationship with each other. However, change in the variables was analyzed as occurring in parallel rather than testing whether improvement in one leads to improvement in the other. The focus of this study was to test whether the trial authors’ theory of how the trial worked was correct rather than test a more complex model of multiple mediating factors. The goodness of fit statistics provided for the mediation model presented previously did not all meet the criteria for good fit, indicating that the hypothesized pathways may not be the only explanation for the effect of treatment on change in disability. Future research could test a more complicated model that includes multiple potential mediators in a single pathway, to show a process of change in several variables as part of the treatment process. This would also allow testing of other variables that might be important in leading to a change in outcome, such as treatment expectations, that may help to further explain how the treatment worked.

### Comparison With Previous Findings

Few studies of treatment mediation in musculoskeletal pain populations have been conducted to date.^33^ This analysis therefore adds important information to the current evidence base. Two previous studies of treatment mediation in primary care LBP populations^45^, ^46^ have also reported evidence for psychological factors being mediators of treatment effect, but these studies used more intensive, CBT-based therapies. The STarT Back intervention was deliberately designed to be a less intensive “light-touch” therapy that could still address psychological factors. The studies also used different methods of mediation analysis and did not include latent variables, making it difficult to compare their results with those found in the present analysis.

### Potential Limitations

The full information maximum likelihood (sensitivity) analysis and nonresponse analysis suggested that the participants included in the complete case analysis differed substantially from those who were excluded, indicating high risk of attrition bias. This was particularly apparent in the psychological measures, in which responders at 4-month follow-up, particularly in the control group, had lower baseline scores of fear-avoidance beliefs, catastrophizing, and depression compared with nonresponders. This means that the results presented in this report need to be interpreted with caution, because they represent only a selective subsample of the high-risk population included in the trial.

Pain duration was not accounted for in this mediation analysis. Different levels of pain duration may have affected patients in different ways; patients with new pain episodes are likely to have lower levels of catastrophizing thoughts or fear-avoidance beliefs, whereas patients with persistent pain may have higher levels of distress. One way of testing this would be to carry out a moderated mediation analysis.^2^ However, it was believed that subgrouping patients further when already investigating only the high-risk subgroup would have affected the reliability and robustness of the analysis.

Temporality, or the order in which change occurred, is a major issue in mediation analysis because all mediation models, regardless of analysis method used, assume a causal order to the variables. However, this causal order is rarely tested because studies often do not take enough assessments of the mediator and outcome to establish which of the variables changed first. The current study analyzed the association of change between the mediator and outcome variables at the same time points, and it is therefore possible that a reduction in disability could have led to a reduction in pain-related distress, rather than change in distress leading to a change in disability as hypothesized in the present analysis. Additional measurements of all variables of interest would therefore help to establish when change occurs and the order in which it occurs.^25^, ^27^ The STarT Back study did collect data at a long-term follow-up point (12 months), meaning that this time point could have been potentially used in the analysis to examine change in outcome over a longer time period. However, the aim of this mediation analysis was to investigate what could have been responsible for the effect of the intervention on outcome, and investigating change over a longer period of time may mean that other, external factors could have affected change in the outcome. This further highlights the issue of when measures should be taken to map the mediating pathway.

It should be noted that the 2 latent variables of pain intensity and pain-related distress were highly correlated, suggesting that multicollinearity may be an issue in this analysis. This was acknowledged in the SEM by allowing the 2 constructs to covary. Pain and distress are closely interlinked and we believed that separating the 2 concepts or looking at them in isolation would not provide an adequate test of the study hypothesis.

### Clinical Implications

Exploring mediators of treatment effect has important implications for clinical practice in that identifying the key factors that lead to improved outcomes will help lead to more focused interventions by providing information on the parts of treatment that are key to changing outcome. This is important because many interventions for LBP are multifaceted and it is unclear which of the different treatment components are necessary for patients to improve. Although this analysis investigates a broad psychological factor, future studies could investigate more specific factors in a more focused intervention, or other modifiable, nonpsychological factors deemed important in the process of change in outcome. More streamlined treatments may reduce the number or length of treatment sessions needed, thereby also reducing treatment costs. This study represents a first step in this process, and more evidence is required for factors to be more definitively found to be on the causal pathway.

### Conclusions

The psychological variables that physiotherapists aimed to address during the STarT Back high-risk intervention explained a significant proportion of the treatment effects observed in the trial. The mediation analysis conducted represents a robust analysis of potential treatment mediators and emphasizes the importance for intervention studies to be underpinned by a clear theoretical or conceptual model. However, this analysis only investigated change between 2 time points, which was not enough to assess the temporal order of the variables in the model. Trials that are designed to adequately test for mediating effects, with variables being measured at appropriate time points during treatment, are required to provide stronger evidence of treatment mediation.

## Figures and Tables

**Table 1:** Baseline Means and SDs and Mean Change at 4-Month Follow-Up for Potential Mediator Variables in the STarT Back Data Set High-Risk Group

	Baseline Score, Mean (SD)		Four-Month Follow-Up, Mean Change (SD)	
	High-Risk Treatment Group (n = 93)	High-Risk Control Group (n = 45)	High-Risk Treatment Group (n = 93)	High-Risk Control Group (n = 45)
Outcome				
Disability	14.41 (4.31)	14.07 (4.88)	7.49 (6.48)	3.62 (4.38)
Potential mediators				
Catastrophizing thoughts	25.24 (1.49)	25.88 (1.54)	11.07 (12.95)	6.64 (1.26)
Fear-avoidance beliefs	46.21 (5.17)	45.52 (5.85)	9.24 (7.56)	3.40 (4.68)
Anxiety	1.01 (4.39)	1.31 (3.59)	3.39 (4.10)	2.49 (3.95)
Depression	8.77 (4.34)	8.40 (3.70)	3.55 (4.05)	1.69 (3.55)
Pain intensity				
Least	6.16 (2.58)	5.96 (3.25)	2.98 (2.87)	1.76 (3.19)
Average	7.72 (2.12)	8.18 (1.80)	3.90 (3.24)	2.56 (2.62)
Current	6.40 (2.33)	6.51 (2.64)	3.00 (2.88)	1.62 (3.00)

**Table 2:** Univariable Associations of Changes in Each Potential Mediator With Change in Functional Outcomes in STarT Back Participants: Linear Regression Analyses

Outcome	Predictor	Treatment Allocation	Change at 4-Month Follow-Up			
			Unstandardized B (Standard Error)	95% CI	Standardized β	R^2^ Change
RMDQ^Δ^	Catastrophizing thoughts^Δ^	Treatment (n = 93)	.49 (.09)	.31–.67	.50	.25
	Fear-avoidance beliefs^Δ^		.57 (.09)	.40–.74	.57	.33
	Anxiety^Δ^		.59 (.09)	.40–.77	.56	.31
	Depression^Δ^		.66 (.09)	.48–.83	.62	.39
	Least pain^Δ^		.77 (.08)	.62–.93	.72	.51
	Average pain^Δ^		.77 (.07)	.63–.91	.74	.55
	Current pain^Δ^		.84 (.07)	.71–.98	.80	.63
	Catastrophizing thoughts^Δ^	Control (n = 45)	.58 (.11)	.37–.80	.64	.41
	Fear-avoidance beliefs^Δ^		.73 (.12)	.05–.97	.67	.45
	Anxiety^Δ^		.46 (.10)	.27–.65	.59	.35
	Depression^Δ^		.58 (.09)	.40–.75	.71	.50
	Least pain^Δ^		.45 (.10)	.26–.65	.59	.34
	Average pain^Δ^		.50 (.11)	.28–.72	.57	.32
	Current pain^Δ^		.47 (.10)	.27–.68	.58	.34

NOTE. ^Δ^indicates residualized change.

**Table 3:** Univariable Associations of Change in Treatment Allocation With Change in Each Potential Mediator: Linear Regression Analyses

Outcome	Predictor	Unstandardized B (Standard Error)	95% CI	Standardized β	R^2^ Change
Catastrophizing thoughts^Δ^	Treatment allocation	.41 (.18)	.06–.76	.19	.04
Fear-avoidance beliefs^Δ^	Treatment allocation	.72 (.17)	.39–1.06	.34	.12
Anxiety^Δ^	Treatment allocation	.28 (.18)	−.08 to .63	.13	.02
Depression^Δ^	Treatment allocation	.47 (.18)	.12–.82	.22	.05
Least pain^Δ^	Treatment allocation	.44 (.18)	.08–.79	.21	.04
Average pain^Δ^	Treatment allocation	.58 (.18)	.23–.92	.27	.07
Current pain^Δ^	Treatment allocation	.52 (.18)	.17–.87	.25	.06

NOTE. ^Δ^indicates residualized change.

**Table 4:** Total, Direct, and Indirect Effects of the Mediation Model on Change in Disability for High-Risk Patients

Effect	Model	
	Standardized Estimates (95% CI)	Unstandardized Estimates (95% CI)
RMDQ^Δ^		
Total (*ć*)	.30 (.14–.43)	.63 (.29–.94)
Direct (*c*)	.05 (−.05 to .16)	.11 (−.11 to .33)
Indirect (*ab*)	.25 (.09–.39)	.52 (.19–.85)

Abbreviations: *ć*, total path of the whole model; *c*, direct effect of treatment on outcome; *ab*, product of coefficients.

NOTE. ^Δ^indicates residualized change.

## Supplementary Data

Supplementary Appendix 1 Supplementary Appendix 1

Supplementary Section 1 Supplementary Section 1
