# Supplementary material for: Exploring What Factors Mediate Treatment Effect: Example of the STarT Back Study High-Risk Intervention
Source: J Pain. 2016 Nov;17(11):1237–45. doi: 10.1016/j.jpain.2016.08.005 (PMC5123895; doi:10.1016/j.jpain.2016.08.005)
Supplement: Supplementary Appendix 1 [file mmc1.docx]

**Appendix 1 Full analysis of High-risk Group using all available data (FIML)**

*Preliminary analysis*

|  | | **Baseline Score (Mean and SD)** | | **Four-month follow-up (Mean change and SD)** | |
| --- | --- | --- | --- | --- | --- |
|  |  | **High-risk treatment group**  **(*n*=157)** | **High-risk control group**  **(*n*=79)** | **High-risk treatment group**  **(*n*=131)** | **High-risk control group**  **(*n*=56)** |
| ***Outcome*** | | | | | |
| Disability | | 14.01 (4.64) | 14.72 (4.40) | 6.77 (6.81) | 4.21 (5.03) |
| ***Potential Mediators*** | | | | | |
| Catastrophising thoughts | | 26.41 (10.63) | 26.89 (10.27) | 10.33 (12.36) | 6.82 (10.41) |
| Fear-avoidance beliefs | | 45.81 (5.04) | 45.96 (5.65) | 8.55 (7.44) | 3.67 (4.65) |
| Anxiety | | 10.11 (4.15) | 10.09 (3.77) | 3.00 (3.95) | 2.46 (3.90) |
| Depression | | 8.90 (4.32) | 8.91 (3.68) | 3.13 (3.96) | 1.56 (3.38) |
| Pain Intensity | Least | 6.33 (2.56) | 5.84 (3.04) | 2.96 (2.87) | 1.88 (3.29) |
|  | Average | 7.94 (1.93) | 8.05 (1.76) | 3.87 (3.20) | 2.51 (2.93) |
|  | Current | 6.68 (2.20) | 6.58 (2.40) | 2.81 (2.81) | 1.47 (2.89) |

*Testing Criteria for Mediation – Linear regressions to show associations between change in potential mediator and change in outcome (b path)*

| **Outcome** | **Predictor** | **Treatment Allocation** | **Change at 4 month follow-up** | | | |
| --- | --- | --- | --- | --- | --- | --- |
|  |  |  | **Unstandardised B (SE)** | **95% CI** | **Standardised Β** | **R-square change** |
| **RMDQ^∆^** | Catastrophising thoughts^∆^ | Treatment (*n*=157) | 0.54 (0.07) | 0.40 to 0.67 | 0.59 | 0.34 |
|  | Fear-avoidance beliefs^∆^ |  | 0.58 (0.08) | 0.42 to 0.74 | 0.57 | 0.32 |
|  | Anxiety^∆^ |  | 0.56 (0.08) | 0.40 to 0.73 | 0.55 | 0.30 |
|  | Depression^∆^ |  | 0.58 (0.08) | 0.43 to 0.73 | 0.59 | 0.35 |
|  | Least pain^∆^ |  | 0.86 (0.09) | 0.69 to 1.03 | 0.69 | 0.47 |
|  | Average pain^∆^ |  | 0.88 (0.08) | 0.72 to 1.04 | 0.72 | 0.52 |
|  | Current pain^∆^ |  | 1.00 (0.07) | 0.85 to 1.14 | 0.79 | 0.62 |
|  | Catastrophising thoughts^∆^ | Control (*n*=79) | 0.53 (0.09) | 0.35 to 0.71 | 0.64 | 0.41 |
|  | Fear-avoidance beliefs^∆^ |  | 0.63 (0.13) | 0.37 to 0.88 | 0.58 | 0.33 |
|  | Anxiety^∆^ |  | 0.48 (0.11) | 0.27 to 0.69 | 0.54 | 0.29 |
|  | Depression^∆^ |  | 0.57 (0.11) | 0.34 to 0.80 | 0.58 | 0.34 |
|  | Least pain^∆^ |  | 0.64 (0.12) | 0.40 to 0.87 | 0.62 | 0.38 |
|  | Average pain^∆^ |  | 0.70 (0.13) | 0.45 to 0.96 | 0.62 | 0.38 |
|  | Current pain^∆^ |  | 0.57 (0.12) | 0.33 to 0.82 | 0.55 | 0.30 |

*^∆^=residualised change*

*Testing Criteria for Mediation – Linear regressions to show associations between treatment allocation and change in potential mediator (a path)*

| **Outcome** | **Predictor** | **Unstandardised B (SE)** | **95% CI** | **Standardised Β** | **R-square change** |
| --- | --- | --- | --- | --- | --- |
| Catastrophising thoughts^∆^ | Treatment Allocation | 0.19 (0.22) | -0.25 to 0.63 | 0.07 | 0.00 |
| Fear-avoidance beliefs^∆^ | Treatment Allocation | 0.23 (0.19) | -0.15 to 0.60 | 0.10 | 0.01 |
| Anxiety^∆^ | Treatment Allocation | 0.23 (0.18) | -0.17 to 0.62 | 0.09 | 0.01 |
| Depression^∆^ | Treatment Allocation | 0.04 (0.20) | -0.35 to 0.43 | 0.02 | 0.00 |
| Least pain^∆^ | Treatment Allocation | 0.38 (0.17) | 0.05 to 0.70 | 0.18 | 0.03 |
| Average pain^∆^ | Treatment Allocation | 0.52 (0.16) | 0.20 to 0.84 | 0.25 | 0.06 |
| Current pain^∆^ | Treatment Allocation | -.40 (0.16) | 0.18 to 0.81 | 0.23 | 0.05 |

*^∆^=residualised change*

High-risk group

FIML (*n*=236)

0.70**

0.04

0.47**

0.20*

0.51**

-0.13*

Disability^∆^

Treatment (Intervention/Control)

-0.02*

*^∆^residualised change*

**p<0.05*

***p<0.01*

*All values are standardised*

Total, Direct and Indirect Effects of each Potential Mediator on Change in Disability for High Risk Patients

|  | Effect | Model | |
| --- | --- | --- | --- |
|  |  | Standardised | Unstandardised |
| RMDQ^∆^ | Total | -0.02 | -0.04 |
|  | Direct | -0.13 | -0.33 |
|  | Indirect | 0.11 | 0.29 |

*^∆^residualised change*

**p<0.05*

Model Fit Statistics for Mediation Model of Change in Disability for High Risk Patients

| Model Index | Current Model | Good Model Fit |
| --- | --- | --- |
| CMIN* | 44.67 | Non-significant result |
| DF | 23 |  |
| P | 0.00 |  |
| CMIN/DF | 1.94 | Between 2-5 |
| CFI | 0.97 | Closer to 1 |
| RMSEA | 0.06 (0.04 to 0.09, PCLOSE 0.20) | <0.08 |
| SRMR | - | <0.08 |
