# Supplementary material for: Exploring What Factors Mediate Treatment Effect: Example of the STarT Back Study High-Risk Intervention
Source: J Pain. 2016 Nov;17(11):1237–45. doi: 10.1016/j.jpain.2016.08.005 (PMC5123895; doi:10.1016/j.jpain.2016.08.005)
Supplement: Supplementary Section 1 [file mmc2.docx]

**Supplement 1: Outline of Factor Analysis**

***Methods***

As Structural Equation Modelling (SEM) allows for the inclusion of latent variables, exploratory factor analysis (EFA) and subsequently confirmatory factor analysis (CFA) were used to examine whether the four psychological constructs tested as mediators were representative of a single latent factor of pain-related distress. EFA was also conducted on the pain intensity measures to see if they represented a single factor of ‘low back pain’.

The statistics of interest when performing EFA are the Kaiser-Meyer-Olkin (KMO) statistic (Kaiser 1970) was tests whether the number of cases was adequate for the analysis to be performed, and the Bartlett’s test of sphericity which tests whether the correlations were too low for the analysis to be valid (Field 2009). The KMO statistic produces a value between 0 and 1, with values of 0.70 or above seen as showing an acceptable number of cases for analysis (Field 2009), while a statistically significant Bartlett’s test indicates that correlations between the items are large enough for the factor analysis to be robust. The EFA models were fitted using a maximum likelihood (ML) procedure as recommended by others (Fabrigar et al 1999; Costello & Osborne 2005). Eigenvalues (a measure of the explained variance of a factor (Kahn 2006)), where values above 1 are generally extracted, and Cattell’s scree plot, in which the eigenvalues are plotted onto a graph in descending order, were used to determine the number of factors to extract.

The EFA was performed on a randomly selected 50% of the data and a confirmatory factor analysis (CFA) was performed on the remaining half, in order to verify the structure created in EFA (Fabrigar et al 1999). The CFA provided model fit statistics to determine whether the proposed latent variable was a good fit to the data.

***Results***

For the EFA of the pain-related distress variable, 394 cases were selected for analysis. The KMO statistic of 0.75 suggested that this was adequate for EFA to be performed. Bartlett’s test also indicated that correlations between variables were high (*X*^2^_(6)_ = 474.57, *p*<0.05). One factor was extracted, confirmed by the scree plot and factor loadings (Table 1), which explained 50% of the variance.

Table 1 Factor matrix (EFA) for the pain-related distress latent variable: Randomly selected 50% of STarT Back population (*n*=394)

| **Measure** | **Factor loading** |
| --- | --- |
| Depression^∆^ (HADS-D) | 0.85 |
| Anxiety^∆^ (HADS-A) | 0.75 |
| Fear-avoidance beliefs^∆^ (TSK) | 0.61 |
| Catastrophising thoughts^∆^ (PCS) | 0.58 |

*^∆^=residualised change*

For the CFA, a model was generated with a single factor, labelled “pain-related distress”. The model fit statistics indicated that overall the model provided an adequate fit to the data (*X*^2^=1.14_(2)_, *p*=0.57; *X*^2^/df=0.57; CFI=1.0, RMSEA=0.00 (95% CI 0.00 to 0.14); SRMR=0.01), suggesting that it was appropriate to use a single pain-related distress factor to represent the four potential psychological mediators.

The analysis was then repeated to create a latent variable of “Low back pain”. In this analysis, the software package selected 588 cases for analysis. This was verified as appropriate by the KMO statistic (0.75) and Bartlett’s test of sphericity was also significant, indicating that the correlations between each scale were adequate (*X*^2^_(3)_ = 1038.707, *p*<0.05). The factor analysis showed that the three measures explain 74% of the variance for the generated factor (confirmed by the scree plot), and the strong correlations in Table 2 below indicate that the three measures represent the extracted factor well.

Table 2 Factor matrix (EFA) for the low back pain latent variable: One-factor extraction model, entire STarT Back population (*n*=588)

| **Measure** | **Factor loading** |
| --- | --- |
| Average Pain^∆^ | 0.90 |
| Current pain^∆^ | 0.84 |
| Least pain^∆^ | 0.84 |

*^∆^=residualised change*
